# ER Stress-induced Inflammasome Activation Contributes to Hepatic Inflammation and Steatosis

**DOI:** 10.4172/2155-9899.1000457

**Published:** 2016-09-26

**Authors:** Jinyu Zhang, Kezhong Zhang, Zihai Li, Beichu Guo

**Affiliations:** 1 Department of Microbiology and Immunology, Medical University of South Carolina (MUSC), Charleston, South Carolina 29425-5040, USA; 2 Hollings Cancer Center, Medical University of South Carolina (MUSC), Charleston, South Carolina 29425, USA; 3 Department of Immunology and Microbiology, Center for Molecular Medicine & Genetics, Karmanos Cancer Institute, Wayne State University School of Medicine, Detroit, MI 48201, USA

**Keywords:** Endoplasmic reticulum, Inflammasome, Steatosis, Liver

## Abstract

Endoplasmic reticulum (ER) stress functions as a protein folding and quality control mechanism to maintain cell homeostasis. Emerging evidence indicates that ER stress is also involved in metabolic and inflammatory diseases. However, the link between ER stress and inflammation remains not well characterized. In this study, we have demonstrated that ER stress-induced inflammasome activation plays a critical role in the pathogenesis of hepatic steatosis. By utilizing genetic and pharmacological agent-induced hepatic steatosis animal models, we found that hepatic steatosis was associated with inflammasome activation and ER stress. Our results show that caspase-1 ablation alleviated liver inflammation and injury. Liver tissues from caspase-1 KO mice had significantly reduced production of IL-1β under ER stress conditions. We also found that ER stress promoted inflammasome activation and IL-1β processing in both hepatocytes and Kupffer cells/macrophages. Moreover, lack of caspase-1 ameliorated cell death or pyropoptosis of hepatocytes induced by ER stress. Taken together, our findings suggest that ER stress-induced inflammasome activation and IL-1β production generate a positive feedback loop to amplify inflammatory response, eventually leading to liver steatosis and injury.

## Introduction

The endoplasmic reticulum (ER) is an intracellular organelle that serves many functions involving in the biosynthesis of membrane and secretory proteins, synthesis of lipids and sterols, and maintenance of intracellular calcium homeostasis [1,2]. ER stress occurs when unfolded and misfolded proteins accumulate in the ER lumen. To maintain ER homeostasis, cells initiate a series of signal transduction pathways collectively termed as Unfolded Protein Response (UPR), which mainly includes inositol-requiring enzyme 1α (IRE1α), double-stranded RNA-dependent protein kinase (PKR)-like ER kinase (PERK), and activating transcription factor-6 (ATF6) pathways [3–5]. While UPR is an adaptive response for stressed cells to adjust translational and transcriptional programs to retain ER homeostasis, prolonged or dysregulated UPR can cause cell death and tissue damage. Numerous studies have demonstrated that ER stress is involved in many diseases including neurodegenerative diseases, metabolic diseases, and inflammatory diseases. However, it remains not fully understood how elevated ER stress contributes to these diseases.

Non-alcoholic fatty liver disease (NAFLD), a spectrum of metabolic disorders ranging from steatosis (NAFL) to steatohepatitis (NASH) to cirrhosis, is the foremost cause of non-alcoholic and non-viral liver-associated illness and death in the developed countries [6]. Hepatic steatosis, characterized by abnormal lipid accumulation, involves a complex network of signaling molecules, lipogenic enzymes and transcription factors [7–9]. Studies have shown that the UPR is activated in several liver diseases; including obesity-associated fatty liver disease [10], viral hepatitis [11], and alcohol-induced liver injury [12], all of which are associated with steatosis, indicating the potential link between ER stress and lipid metabolism.

Inflammasomes are multimolecular complexes, composed of NOD-like protein (NLR) such as NLRP3, the adaptor apoptosis-associated speck-like protein containing a caspase recruitment domain (ASC), and caspase-1 [13–15]. In response to a range of stimuli such as ATP, Alum, or pathogens, NLRP proteins induce the activation of caspase-1, resulting in the cleavage and maturation of inflammatory cytokines pro-IL-1β and pro-IL-18 [16–18]. Individual NLRP, such as NLR3, NLRC4 and NLRP6 can form distinct inflammasomes in response to particular stimuli [17,19]. Recent progress indicates that inflammasomes play a critical role in autoimmune diseases and metabolic syndrome [20–24]. However, whether inflammasome activities promote or protect liver steatosis is still under debate. For example, one report shows that caspase-1 deficiency mice were protected from high fat-induced hepatic steatosis, inflammation and early fibrogenesis [25]. But another study indicates that the NLRP6 and NLRP3 inflammasomes negatively regulated NAFLD/NASH progression [26]. In addition, NLRP3 inflammasome has been shown to contribute to acetaminophen-induced hepatotoxicity [27].

Emerging evidence suggests that hepatic steatosis or fatty liver is frequently associated with a broad array of inflammatory responses and ER stress [24,28–30]. However, the interaction between UPR and inflammasome/IL-1β pathways in liver inflammation and injury remains poorly characterized. In this study, our data show that ER stress-induced inflammasome contributes to the pathogenesis of hepatic steatosis. We found that caspase-1 knockout (KO) mice exhibited much less hepatic steatosis and inflammation. Our results highlight a novel role for the inflammasome activation and IL-1β production in cellular stress-mediated hepatic inflammation and injury.

## Results

### Hepatic steatosis is associated with inflammasome activation and ER stress

**Figure 1:**
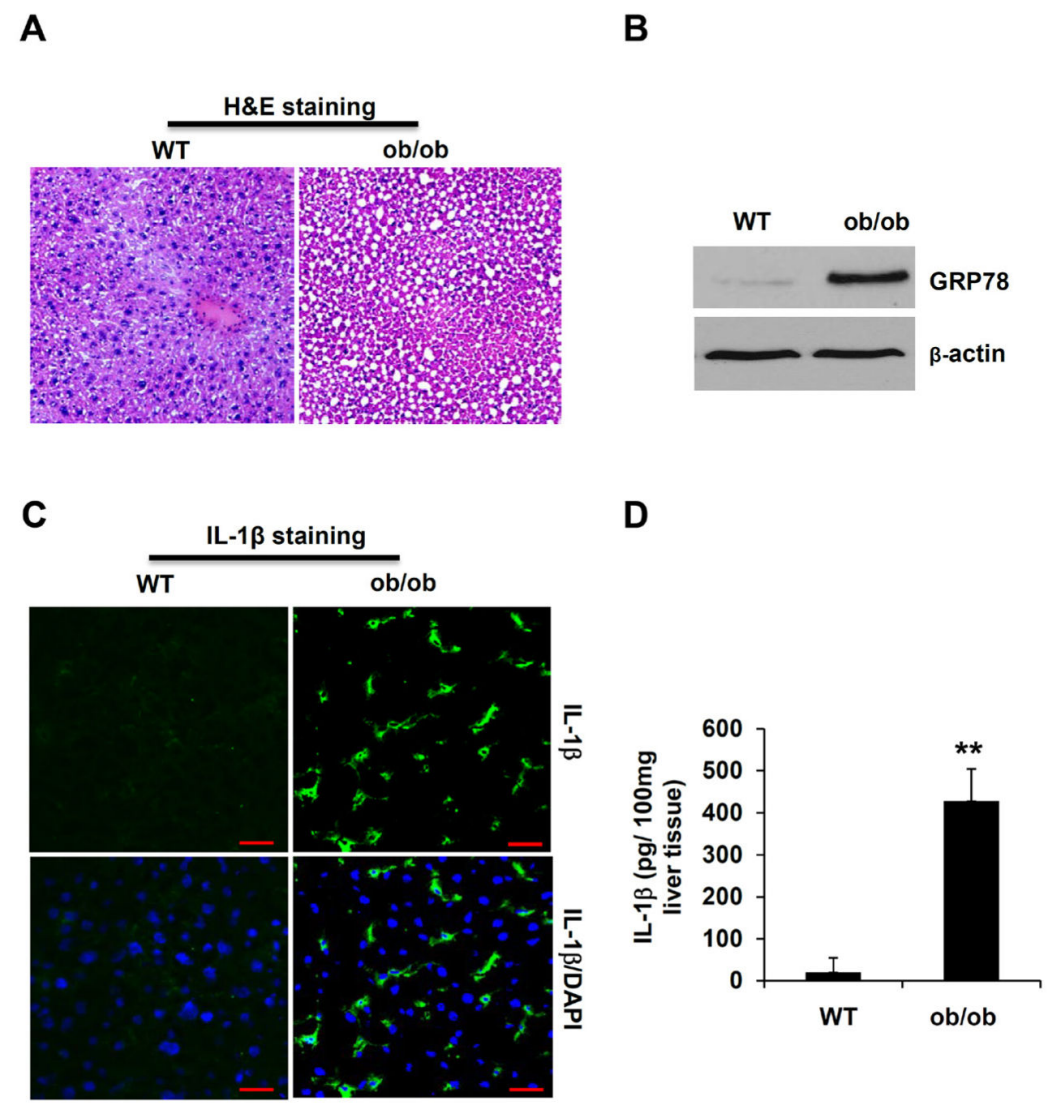
The hepatic steatosis in the liver of ob/ob mice is associated with ER stress and inflammasome activation. **(A)** H&E staining of liver tissue sections from WT and ob/ob mice. **(B)** Liver tissues from WT and ob/ob mice were homogenized, and analyzed by Western Blot for ER stress markers Grp78. β-actin was used as a loading control. **(C)** Liver sections from WT and ob/ob mice were immunostained with an anti-IL-1β specific antibody. The green color indicates IL-1β, and nuclei are stained in blue by DAPI. Scale bar: 100 μm. **(D)** Liver tissues from WT and ob/ob mice were homogenized, and analyzed by IL-1β specific ELISA. Statistical significance is indicated, ^**^P<0.01 (Student’s t test).

To investigate the interaction of inflammasome and ER stress pathways *in vivo*, we utilized ob/ob mice and pharmacological agents-induced steatosis models. ob/ob mice exhibit obesity and diabetes-like syndromes because of spontaneous *leptin* mutation. Those mutant mice also develop fatty liver, therefore are commonly used as a mouse model of NAFLD [30,31]. As expected, ob/ob mice at the age of 10 weeks displayed steatosis phenotype in the liver (Figure 1A). Liver cells from ob/ob mice exhibited ER stress phenotype as indicated by enhanced expression of the ER stress marker GRP78 (Figure 1B). Notably, IL-1β protein staining was significantly increased in liver tissues from ob/ob mice by immunostaining analysis, compared to that from WT mice (Figure 1C). Furthermore, when liver tissues were homogenized and analyzed by IL-1β specific ELISA, liver tissues from ob/ob mice displayed a significant increase in IL-1β protein levels (Figure 1D). Those results imply that liver steatosis is associated with not only ER stress, but also the activation of inflammasome and production of mature IL-1β.

### ER stress-induced liver steatosis is significantly attenuated in caspase-1 KO mice

**Figure 2:**
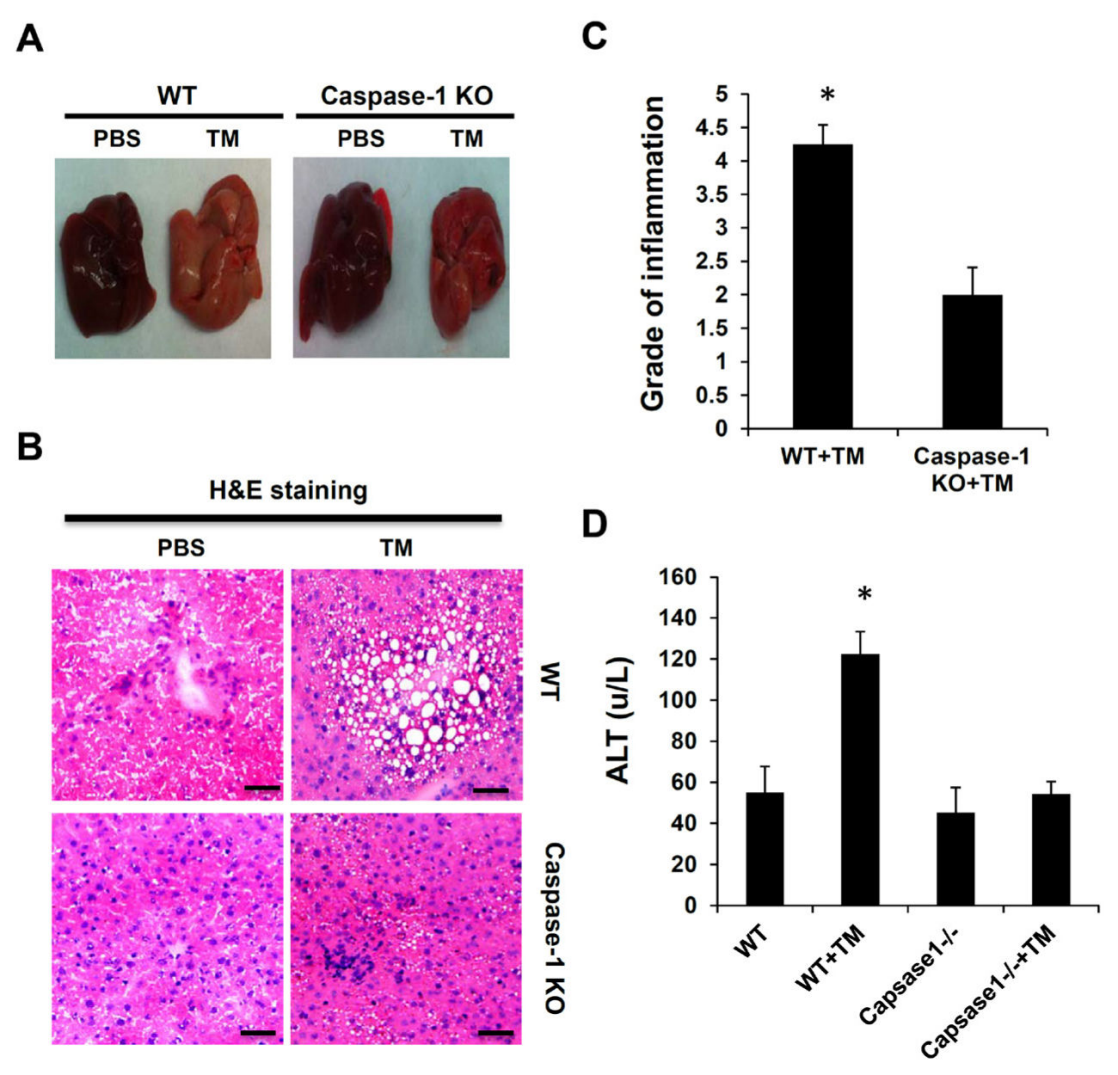
Caspase-1 deficiency attenuates ER stress-induced hepatic steatosis. Mice were intraperitoneally injected with TM (1 mg/kg body weight). **(A)** The morphology of liver, **(B)** H&E staining of liver tissue sections, **(C)** Pathologic inflammation scores, **(D)** plasma Alanine aminotransferase (ALT) level in liver tissues from WT and caspase-1 KO mice treated with or without TM. Each experiment was performed independently at least three times. ^*^P<0.05, ^**^P<0.01 (Student’s t test).

To further define the role of inflammasomes and IL-1β in ER stress-induced hepatic steatosis, we utilized a Tunicamycin (TM)-induced steatosis animal model. TM, an N-linked protein glycosylation inhibitor, can induce robust ER stress response. When injected *in vivo*, it induces hepatic steatosis [32]. WT and caspase-1 KO mice were intraperitoneally (*i.p*) injected with TM. 48 hours post injection liver steatosis and inflammation were examined by hematoxylin and eosin (H&E) staining. Liver function and lipid metabolism were measured by biochemical analysis. As shown in Figure 2, TM treatment in WT mice led to hepatic steatosis accompanied by liver inflammation and damage as indicated by H&E staining and serum ALT assay. Although both groups of mice developed hepatic steatosis, caspase-1 KO mice displayed significantly less hepatic inflammation and steatosis (Figures 2B and 2C). Consistent with the histological results, serum alanine aminotransferase (ALT) level was considerably reduced in caspase-1KO mice (Figure 2D). Furthermore, caspase-1 KO mice showed less body weight loss in response to ER stress, compared to WT mice (Supplementary Figure 1). Those results suggest that the inflammasome activity contributes to ER stress-induced liver steatosis and inflammation.

### ER stress-induced hepatic steatosis and lipogenesis are regulated by the inflammasome pathway

**Figure 3:**
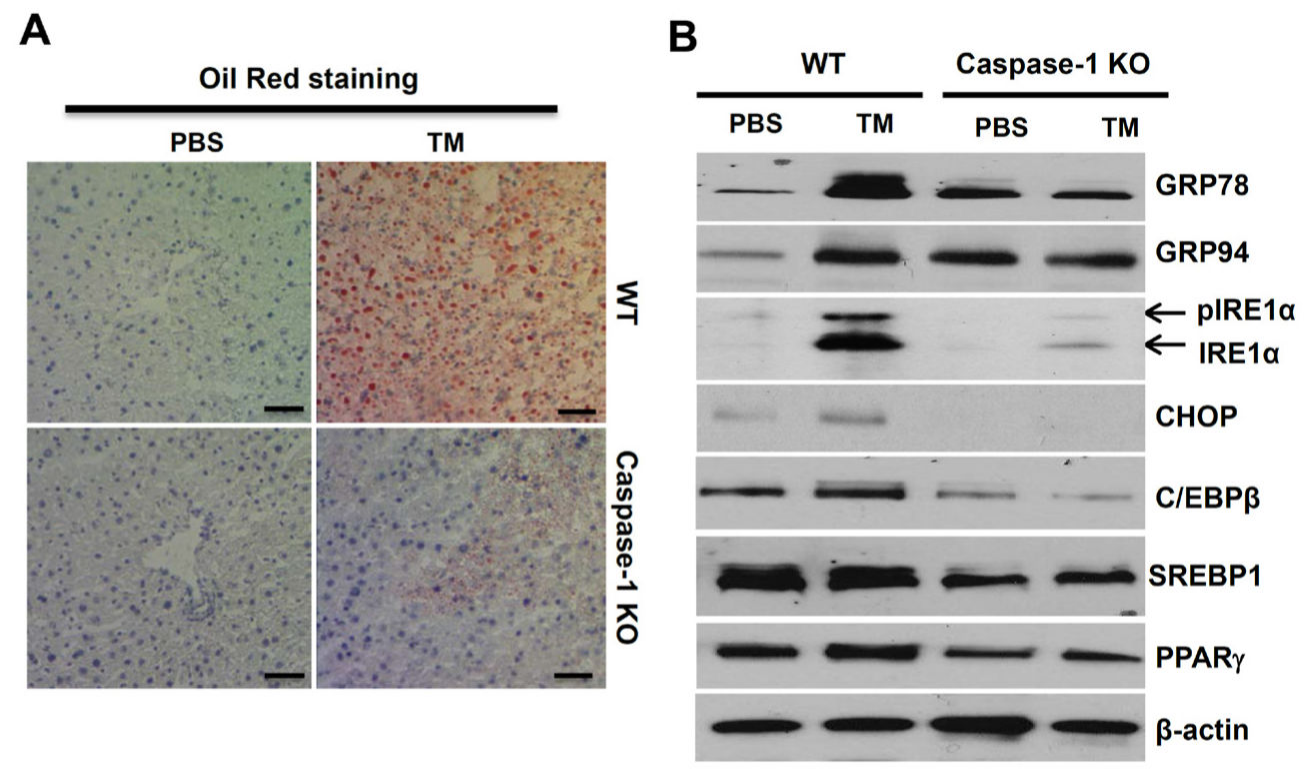
Inflammasomes regulate ER stress-induced lipogenesis. **(A)** Oil red O staining of liver tissue sections from WT and caspase-1 KO mice treated with or without TM. **(B)** Liver tissues from WT and caspase-1 KO mice treated with TM were homogenized, and analyzed by Western Blot for ER stress markers including Grp78, Grp94, CHOP and IRE1α, as well as lipogenic proteins such as C/EBPβ, PPARγ and SEBP1. β-actin was used as a loading control. Each experiment was performed independently at least three times.

To further prove that the inflammasome activity regulates lipid metabolism during ER stress-induced liver steatosis, we performed Oil-Red staining on liver frozen sections. As shown in Figure 3A, WT mice displayed severe fat liver after TM injection, but caspase-1 KO mice had less lipid accumulation. Lipid metabolism and accumulation of fat in the liver are regulated by a number of transcription factors including CCAAT/enhancer binding proteins (C/EBPs), Peroxisome proliferator-activated receptor gamma (PPARγ), and Sterol regulatory element-binding protein 1 (SREBP1) [33,34]. To determine whether inflammasome activities influence lipid metabolism in the liver, we examined the induction of ER stress marks and lipid-related transcriptional factors in liver tissues from WT or caspase-1 KO mice injected with TM. As shown in Figure 3B, TM treatment resulted in a significant increase in ER stress markers, including BIP/GRP78, Grp94, CHOP and IRE1α, as well as transcription factors regulating lipid metabolism in liver tissues of WT mice. Remarkably, the induction of ER stress proteins and lipid-related transcriptional factors was significantly diminished in the liver tissues from caspase-1 KO mice. Collectively, these data suggest that inflammasomes either directly or indirectly regulate UPR response and lipid metabolism during the development of hepatic steatosis.

### ER stress-induced hepatic steatosis is associated with increased inflammasome activation and IL-1β production

**Figure 4:**
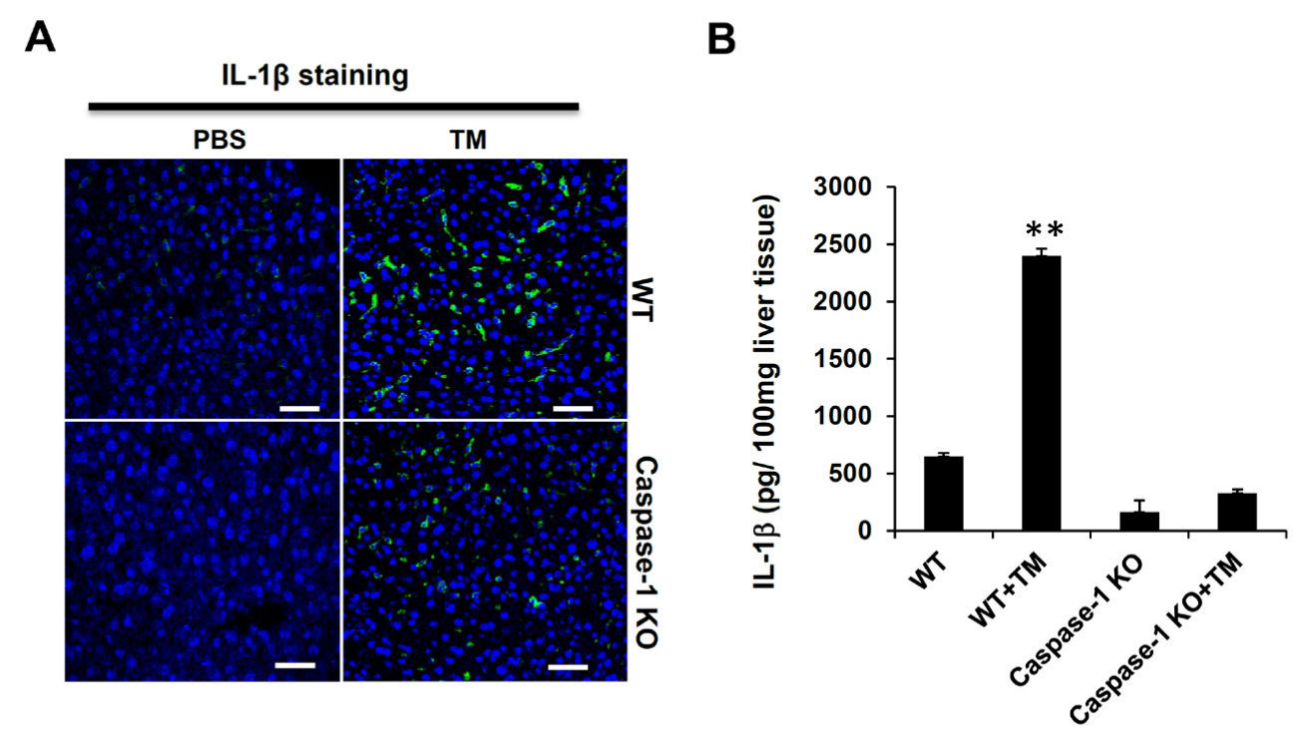
ER stress-induced hepatic steatosis is associated with inflammasome activation and IL-1β production. **(A)** IL-1β immunostaining of liver sections. IL-1β levels in liver tissues from WT and caspase-1KO mice treated with or without TM were detected by immunostaining with an anti-IL-1β specific antibody. The green color indicates IL-1β, and nuclei are stained in blue by DAPI. Scale bar: 100 μm. **(B)** Liver tissues from WT and caspase-1 KO mice treated with TM treatment were homogenized, and analyzed by ELISA. Data are presented as means ± SEM. ^**^P<0.01 (Student’s t test).

Our results suggest that ER stress-induced hepatic steatosis and inflammation are regulated by caspase-1 activity. Thus we examined inflammasome activation in steatotic tissues. IL-1β immunostaining was performed on liver tissues from mice treated with or without pharmacological ER stress inducers. We found that there was very weak IL-1β staining in liver sections from untreated mice, and a significant increase in IL-1β staining in liver tissues from WT mice treated with TM. In contrast, much less IL-1β staining was observed in liver sections from caspase-1 KO mice injected with the ER stress inducer (Figure 4A). Alternatively, we measured IL-1β proteins levels in liver tissues. After TM treatment, liver tissues from WT and caspase-1 KO mice were homogenized and assayed by IL-1β specific ELISA. Consistent with the immunostaining result, we found that IL-1β protein concentration was significantly increased in liver tissue of TM-injected WT mice. But ER stress-induced IL-1β production was abrogated in TM-treated caspase-1 KO mice (Figure 4B).

### ER stress promotes inflammasome activation and IL-1β processing in macrophages

**Figure 5:**
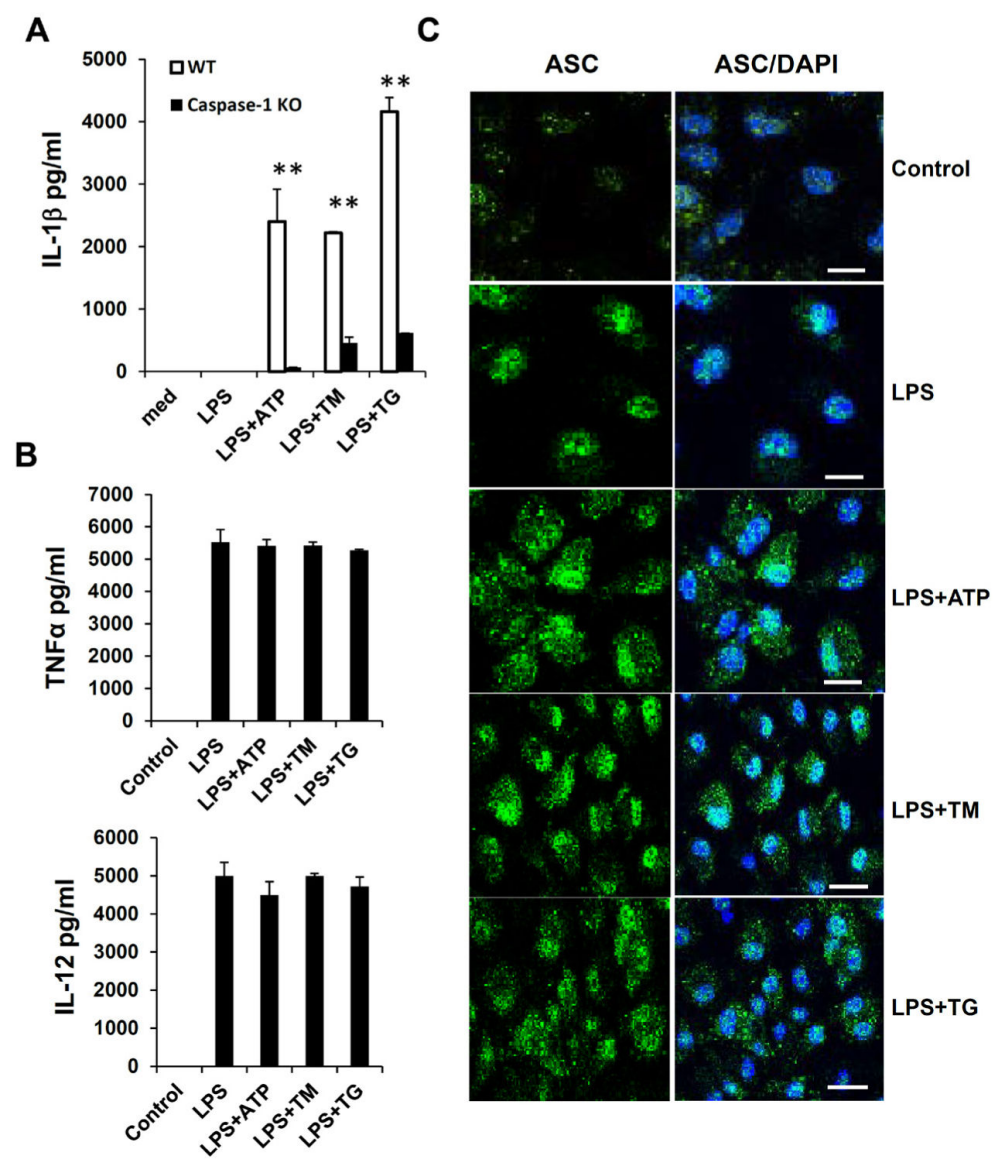
ER stress induces inflammasome activity and IL-1β processing in macrophages. **(A)** Bone marrow-derived macrophages (BMDMs) from WT and caspase-1 KO mice were primed with 250 ng/ml LPS for 4 hr and then treated with 1 mM ATP, 10 μg/ml TM or 5 μM TG. The concentrations of IL-1β in culture supernatants were assayed by ELISA. **(B)** The concentration of TNFα or IL-12 in culture supernatants of WT macrophages treated with LPS and ER stress inducers as in **(A)** was assayed by ELISA respectively. **(C)** Macrophages were treated as in **(A)**, and then NLRP/ASC/caspase-1 complexes were detected using an anti-ASC specific antibody by immunostaining. The green color indicates the expression of ASC protein, and nuclei are stained in blue by DAPI. Scale bar: 100 μm. Data are presented as means ± SEM. ^**^P<0.01 (Student’s t test).

Hepatic steatosis is a complicated process regulated by multiple cell types and signaling pathways in the liver. To prove that ER stress is able to induce inflammasome activation in macrophages or Kupffer cells, we used primary bone marrow-derived macrophages (BMDMs) to test our hypothesis. BMDMs from WT and caspase-1 KO mice were primed with LPS to induce the expression of pro-IL-1β, and then treated with ER stress inducers TM or Thapsigargin (TG) to activate inflammasomes. TG inhibits intracellular Ca^2+^ pump function in the ER, which leads to accumulation of misfolded proteins and UPR [35]. We also used LPS/ATP stimulation as a positive control for inflammasome activation. The IL-1β production from WT and caspase-1 deficient macrophages was measured by ELISA. As shown in Figure 5A, there were no or very low levels of mature IL-1β in untreated cells or macrophages treated with LPS alone. LPS plus TM or TG induced a substantial production of IL-1β from WT macrophages. In contrast, IL-1β secretion was markedly reduced in caspase-1 deficient macrophages treated with LPS/TM or LPS/TG (Figure 5A). In our experimental conditions, the dosage and duration of TM or TG treatment used for inflammasome activation specifically affected IL-1β production, since LPS-induced TNFα or IL-12 production was not affected during the period of TM or TG treatment (Figure 5B). To further support our conclusion, we examined inflammasome activation by ASC immunostaining. When activated, multiple ASC molecules form large signaling complexes with NLRPs and caspase-1, which can be visualized by immunostaining. Compared to LPS stimulation alone, ER stress induced the formation of aggregated dot structure of ASC molecules (Figure 5C).

To elucidate ER stress pathways responsible for inflammasome activation and IL-1β processing, we examined the expressions of ER stress markers including ATF6α, IRE1α, PERK, PKR, BiP and GRP94 in BMDMs treated with LPS and ER stress inducers. We found that inflammasome and ER stress inducers didn’t affect the expression of ATF6α. However, others ER stress markers were significantly increased during inflammasome activation (Supplementary Figure 2A). Furthermore, the spliced XBP-1 mRNA wasn’t detected in BMDMs treated with LPS or LPS/ATP, but detected in BMDMs with TM or TG (Supplementary Figure 2B). These data indicate that ER stress may use different signaling pathways to induce inflammasome activation. Furthermore, we also found that ER stress induced inflammasome activation in human macrophages/monocytes. When THP-1 cells, a human monocyte cell line, were treated with LPS plus ER stress inducers, the IL-1β production was significantly induced (Supplementary Figure 3).

### Caspase-1 deficiency attenuates hepatic cell death induced by ER stress

**Figure 6:**
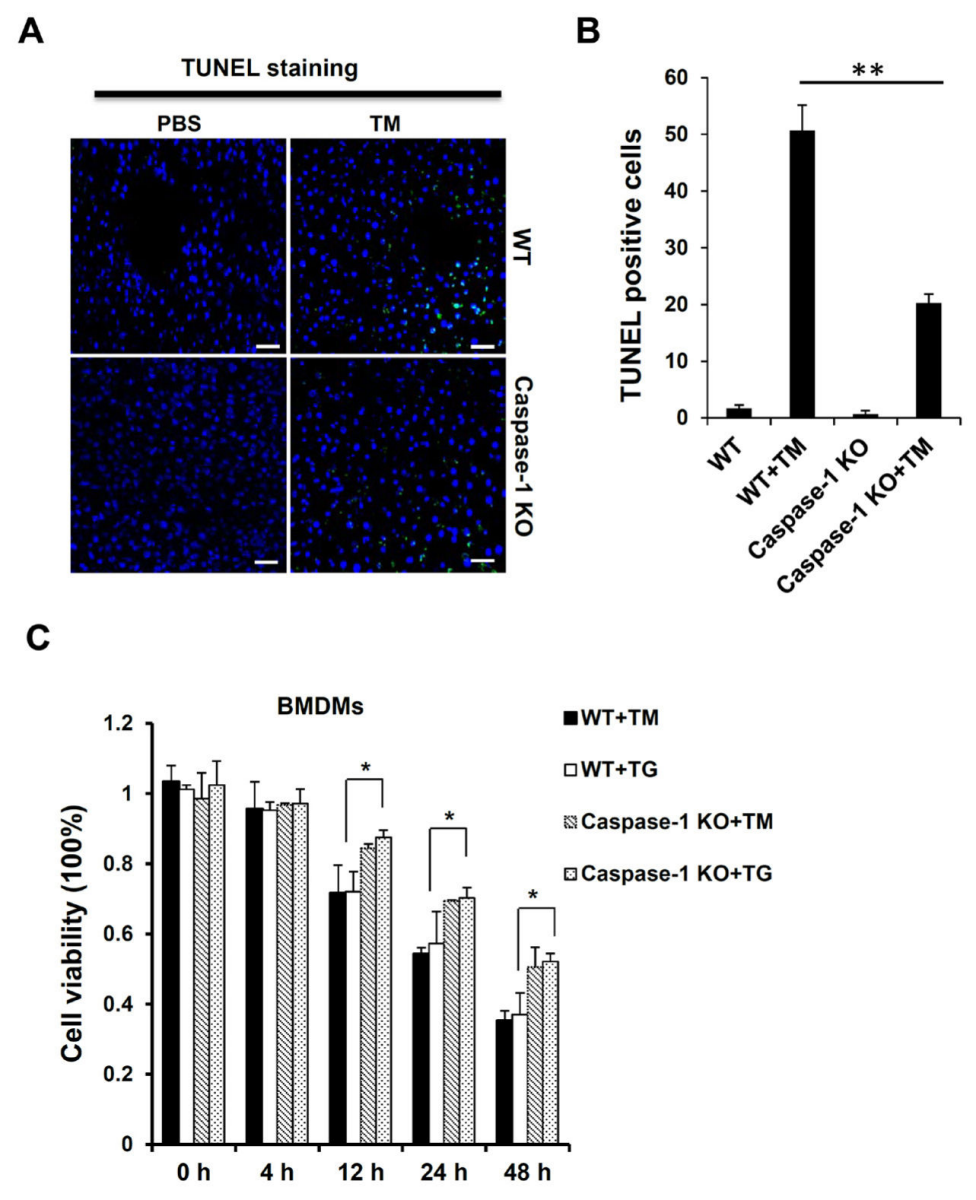
Caspase-1 ablation alleviates cellular pyroptosis induced by ER stress. **(A)** TUNEL staining of liver sections from WT and caspase-1 KO mice injected with TM (n ≥ 5 for each group). The green color indicates TUNEL positive cell, and nuclei are stained in blue by DAPI. **(B)** The percentage of TUNEL positive cells in liver tissues from mice treated in **(A)**. Liver from WT mice treated with TM displayed significantly more TUNEL positive cells throughout the tissue with several dense areas of TUNEL positive cells, indicating pyroptotic cell death. **(C)** Cell viabilities of TM- or TG-treated macrophages from WT and caspase-1 KO mice were examined by MTT (3-(4,5-dimethylthiazol-2-yl)-2,5-diphenyltetrazolium bromide) assay. Data are presented as means ± SEM. ^*^P<0.05. ^**^P<0.01 (Student’s t test).

Inflammasome activation not only induces the production of IL-1β or other cytokines, but also causes pyropotosis, a type of cell death sharing some characteristics of apoptosis and necrosis. We hypothesized that pyropototic cells may contribute to the liver inflammation and damage induced by ER stress. To test this, we performed TUNEL staining on liver tissues. We observed a significant increase in TUNEL positive cells in liver sections of WT mice injected with TM. In contrast, caspase-1 KO mice injected with TM had few TUNEL staining positive cells in the liver (Figures 6A and 6B). To further determine the role of caspase-1 in ER stress-induced cell death, macrophages from WT and caspase-1 KO mice treated with TM or TG were measured for viability by using MTT assay. As shown in Figure 6C, cell death in macrophages could be detected as early as 12 hours after treatment. However, cells from caspase-1 KO mice were more resistant to cell death induced by TG or TM. These results suggest a possible involvement of caspase-1-dependent pyroptosis or cell death pathways in ER stress-induced liver inflammation and injury.

### ER stress induces inflammasome activation in both hepatocytes and Kupffer cells

**Figure 7:**
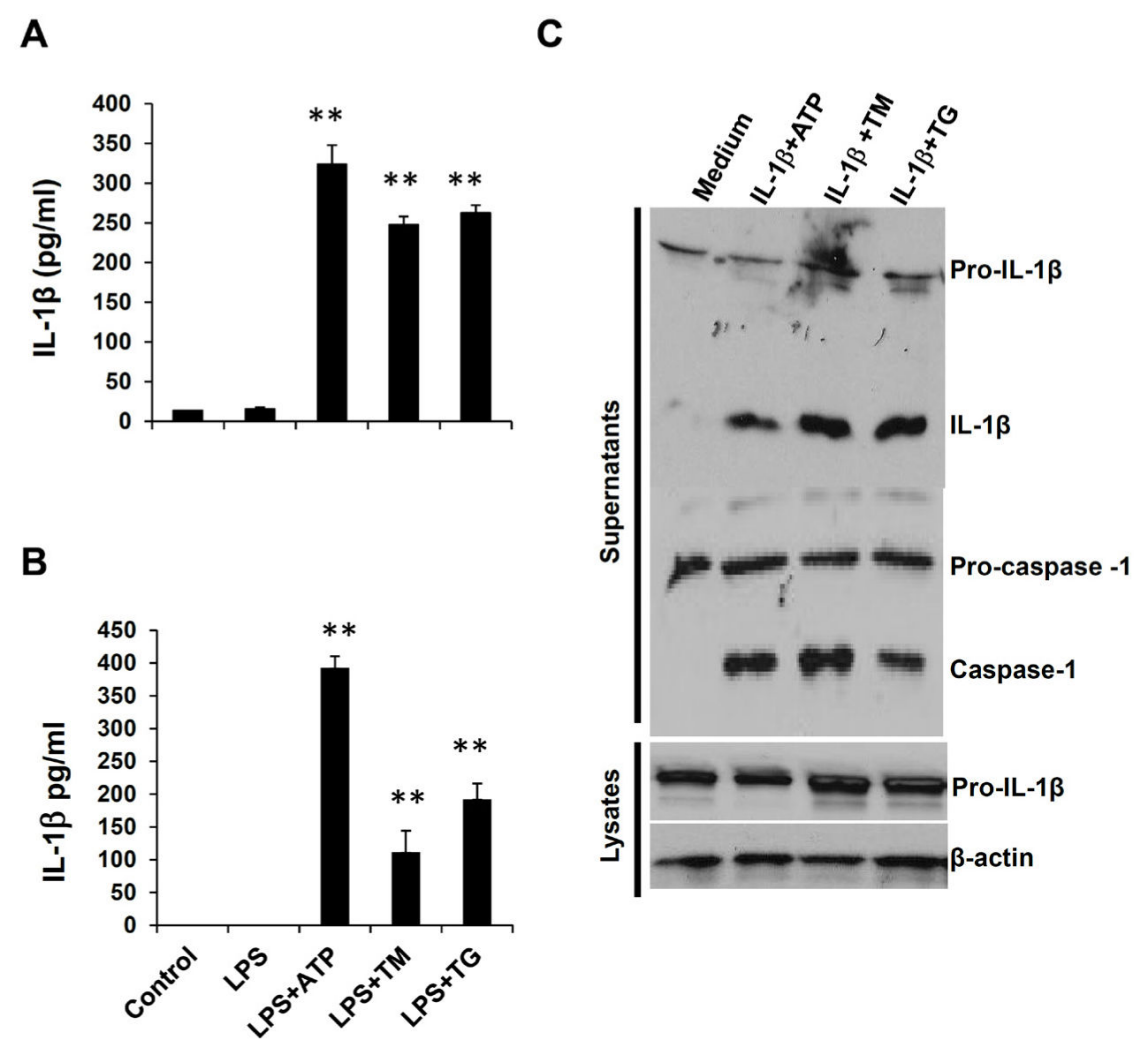
ER stress induces inflammasome activation in both hepatocytes and Kupffer cells. Hepatocytes (A) and Kupffer cells (B) isolated from WT mice were primed with LPS, and then treated with ATP, TM or TG. IL-1β levels in culture supernatants were analyzed by ELISA. Data are presented as means ± SEM. **P<0.01 (Student’s t test). (C) IL-1β amplifies inflammasome activation and its own production. BMDMs from WT mice were pre-treated with IL-1, and then stimulated with ATP, TM or TG, the processing of IL-1β and caspase-1 in culture supernatants and lysates were analyzed by Western Blot.

To identify cell types that undergo inflammasome activation and IL-1β production in response to ER stress, we isolated primary hepatocytes and Kupffer cells, resident macrophages, from liver tissues, and tested inflammasome activation. Interestingly, our results show that both hepatocytes and Kupffer cells were able to produce mature IL-1β after LPS/TM or LPS/TG stimulation (Figures 7A and 7B). These results suggest that ER stress-induced inflammasome activation in both hepatocytes and Kupffer cells could potentially contribute to hepatic steatosis.

Since ER stress-induced steatosis and lipid accumulation were affected by inflammasome activities, we hypothesize that ER stress-induced inflammasome activation and IL-1β regulate liver function and lipid metabolism. To test this, we evaluated if IL-1β plus ER stress inducers can propagate inflammasome activation in macrophages. As shown in Figure 7C, when pretreated with IL-1β, then stimulated with ATP or ER stress inducers, macrophage cells exhibited enhanced inflammasome activities as indicated by pre-IL-1β and pro-caspase-1 processing. These results suggest that ER stress-induced inflammasome activation generates a positive feedback loop to further induce an inflammatory response and modulate lipid metabolism in the liver.

## Discussion

In this study, our results demonstrate that inflammasome plays a critical role in the pathogenesis of hepatic steatosis. We found that ER stress-induced liver steatosis was significantly attenuated in caspase-1 KO mice. Our results show that inflammasomes and IL-1β contributed to ER stress-induced liver inflammation and injury. Furthermore, ER stress induced the inflammasome activation and IL-1β production in both macrophage/Kupffer cells and hepatocytes. However, the molecular pathways that trigger inflammasome activation upon ER stress are poorly understood.

Recent progress indicates that inflammasome activation and IL-1β production are involved in various inflammatory and autoimmune diseases, including liver diseases. For example, IL-1β receptor antagonists ameliorated inflammasome-dependent alcoholic steatohepatitis in mice [36]. Reports also show that NLRP3 inflammasome activation caused liver cell death, severe inflammation and fibrosis [37,38]. Although several studies demonstrated that ER stress induced pro-IL-1β mRNA expression or NLRP3 inflammasome activation, whether classic UPR pathways are involved in inflammasome activation remains controversial [39–41]. For instance, a study from Tschopp’s group has suggested that ER stress triggered NLRP3 inflammasome activation in a mechanism that is independent of the classical ER stress signaling pathways [39], whereas other groups suggested that ER stress-induced inflammasome activation led to β-cell death through mechanisms of TXNIP mRNA stability. In contrast, Lebeaupin et al. did not observe any variation of TXNIP protein expression during ER stress [38]. In addition to NLRP3, other inflammasomes have been identified recently [15,16,42,43]. It will be interesting to know whether ER stress can activate multiple inflammasomes in liver tissues. Thus, further studies are needed to define the NLRP proteins involved and signal pathways connecting UPR and inflammasomes during ER stress-induced hepatic steatosis.

In this study, our results demonstrate that ER stress-induced liver steatosis was significantly attenuated in caspase-1 KO mice. We also demonstrated that the inflammasome/IL-1β pathway, in turn, promoted hepatocytes to express both ER stress markers and transcription factors critical for inflammation and lipid metabolism. Therefore, our studies link classic ER stress pathway to the inflammasome activation in the development of hepatic steatosis. Interestingly, our studies also revealed that IL-1β induced inflammasome activation, promoting its own production in macrophages during ER stress. Those results indicate that ER stress-induced inflammasome activation generates a positive feedback loop, eventually leading to liver steatosis. Our results also show that caspase-1 influenced the expression of transcription factors involved in lipid metabolism, such as PPARγ and SREBP1. Further experiments are needed to elucidate how IL-1R signaling network regulates those transcription factors and lipogenesis.

Although this and other studies indicate the involvement of inflammasome and IL-1β in liver injury and steatosis, the cellular source and targeting cells of IL-1β in hepatic steatosis are less clear. Our results show that both hepatocytes and Kupffer cells underwent inflammasome activation and produced IL-1β after ER stress. We are currently do not know the relative contribution of IL-1β produced from hepatocytes and Kupffer cells, as well as innate immune cells recruited from bone marrow. Studies with cell-specific inflammasome KO or IL-1R KO are required to address those questions. The IL-1 family has at least 11 members [44–46]. While IL-1α and IL-1β play a pivotal role in inflammation and inflammatory diseases, including hepatic steatosis, other members of this family may hold anti-inflammatory or pro-inflammatory functions during liver inflammation and steatosis. Thus, understanding the diverse functions of different IL-1 family members in liver steatosis may help to identify additional treatment for liver diseases. As inflammasome activation and IL-1β play a critical role in the development of hepatic inflammation and steatosis, blocking the inflammasome pathway or IL-1/IL-1R pathways could represent a novel approach for the treatment of liver inflammation and injury. For instance, IL-1R antagonist (IL-1Ra) will be potentially an attractive candidate, which has been used clinically to treat certain autoimmune diseases [47,48].

**Figure 8:**
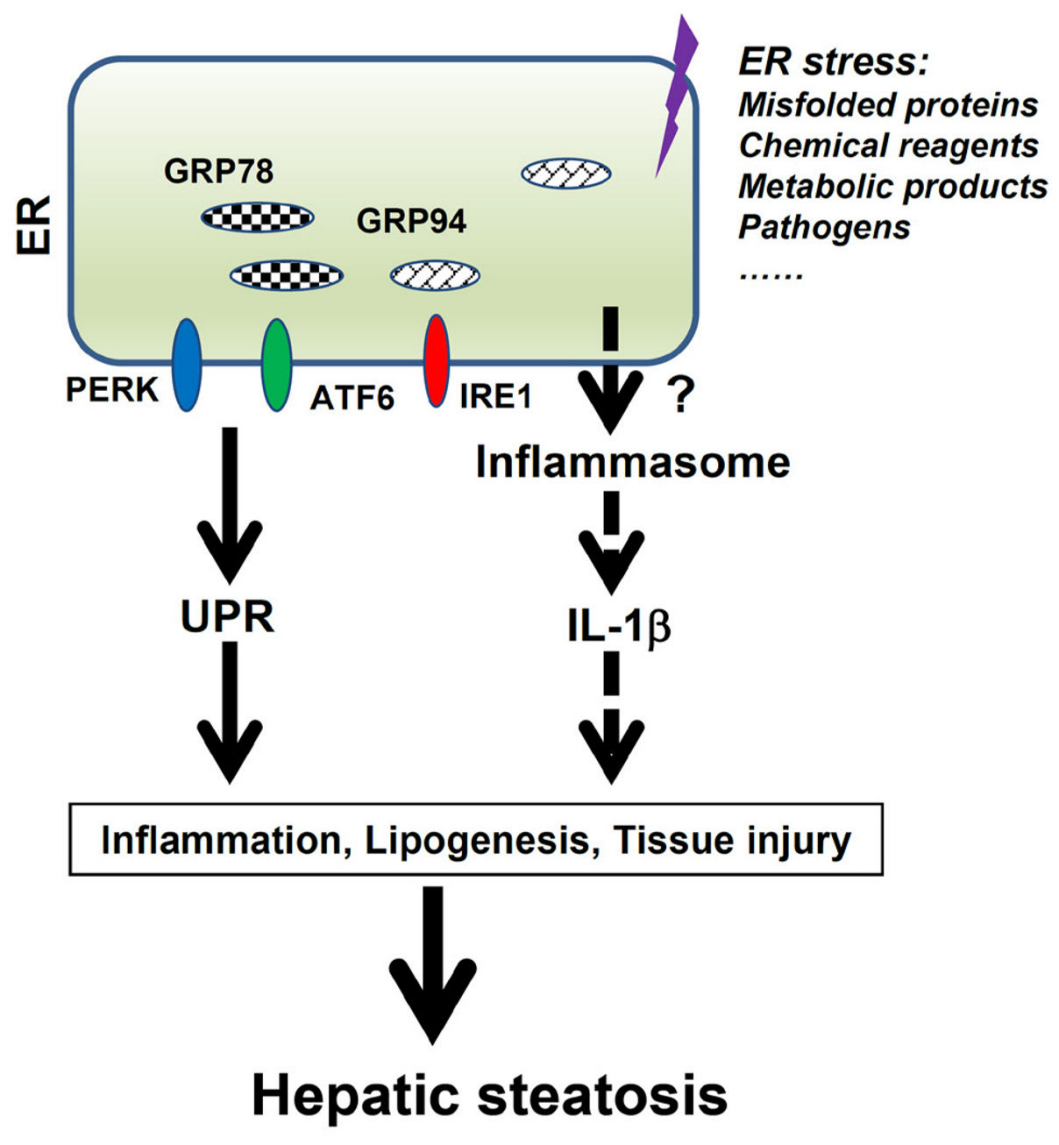
ER stress induces inflammasome activation. ER stress triggers the Unfolded Protein Response (UPR), which is an adaptive response for stressed cells to retain ER homeostasis. However, dysregulated UPR can cause cell death and tissue damage. ER stress potentially induces the activation of inflammasomes and production of IL-1β, which in turn further exacerbates ER stress and inflammation. Inflammasome activation, together with UPR response, contributes to liver damage and steatosis.

In summary, the present study has uncovered the new roles of inflammasome activation and IL-1 production in liver inflammation and damage. Our findings support a model in which inflammasome activation in hepatocytes and Kupffer cells results in the induction of pro-inflammatory signaling and hepatocyte pyroptotic cell death, which contributes to the pathogenesis of hepatic steatosis (Figure 8). While our studies show that ER stress induces inflammasome activation in macrophages and hepatocytes, the molecular mechanisms responsible for inflammasome activation remain unknown. Investigation of the crosstalk between ER stress and inflammasome will help us to develop therapies for the pathogenesis of hepatic steatosis as well as other metabolic and inflammatory diseases.

## Material and Methods

### Reagents and mouse

All chemicals used in this study were purchased from Sigma (St. Louis, MO), unless otherwise noted. Ultrapure LPS, ATP, caspase-1 inhibitor ZVAD-FMK were purchased from Invivogen (San Diego, CA). Mouse IL-1β from PeproTech (Rocky Hill, NJ); human or mouse IL-1β ELISA kits were from R&D system (Minneapolis, MN) or eBioscience (San Diego, California); Antibodies to mouse IL-1β and mouse caspase-1 were from R&D Systems or Santa Cruz Biotechnology (Santa Cruz, CA).

Wild type (WT) C57BL/6, caspase-1 KO and ob/ob (B6.Cg-*Lep^ob^*/J) mice were purchased from the Jackson Laboratory (Bar Harbor, Maine). All mice were maintained at MUSC Hollings animal facility under specific pathogen-free conditions. All animal experiments were approved by the Institutional Animal Care & Use Committee (IACUC) at MUSC, and were conducted in accordance with federal regulations as well as institutional guidelines and regulations on animal studies.

### Hepatic steatosis induction

WT and caspase-1 deficient mice of eight weeks old were injected with TM diluted in DMSO. Tunicamycin (TM) was administrated intraperitoneally at 1 mg/kg mouse weight based on published papers and our previous works [49–53]. 48 hours after injection, blood samples were collected, serum was prepared and liver steatosis was analyzed by staining.

### Bone marrow-derived macrophages (BMDMs)

BMDMs were differentiated as previously described [23]. Briefly, mouse bone marrow cells were cultured for 7 days in DMEM containing 10% fetal bovine serum, penicillin, streptomycin, and 20% conditioned media from L929 cells containing macrophage colony-stimulating factor (M-CSF). To induce IL-1β processing and production, differentiated BMDMs were primed with 250 ng/ml ultrapure LPS for 4 hr, then cells were stimulated with ATP (1 mM) for 1 hr, or TM (10 μg/ml), TG (5 μM) for 4 hr.

### Western blot and ELISA

Cell lysates, cell culture supernatants, or tissues homogenates were analyzed by SDS-PAGE and Western Blot with specific antibodies as described [23,54]. The concentrations of cytokines, including IL-1β and TNF-α, in culture supernatants or tissue samples were detected by ELISA kits (from eBioscience or R&D) according to the manufacturer’s protocol.

### Immunohistochemistry

Approximately 1 cm segments of tissue were embedded in OCT freezing medium (Thermo Scientific) and immediately frozen on dry ice or fixed in 4% formaldehyde/PBS, before embedding in OCT medium. Samples were stored at −80°C until sectioning. 7 μm sections were cut with a cryostat onto poly-L lysine-coated slides (Sigma). For histological examination, all slides were immediately stained with hematoxylin/eosin (H&E), Oil red O staining or immunostaining.
